# Biological and Psychological Perspectives of Resilience: Is It Possible to Improve Stress Resistance?

**DOI:** 10.3389/fnhum.2018.00326

**Published:** 2018-08-21

**Authors:** Haoran Liu, Chenfeng Zhang, Yannan Ji, Li Yang

**Affiliations:** ^1^School of Psychology, Center for Studies of Psychological Application, South China Normal University, Guangzhou, China; ^2^School of Life Sciences, South China Normal University, Guangzhou, China; ^3^Institute for Brain Research and Rehabilitation, South China Normal University, Guangzhou, China

**Keywords:** resilience, stress, depression, VTA, NAc, mPFC

## Abstract

The term “resilience” refers to the ability to adapt successfully to stress, trauma and adversity, enabling individuals to avoid stress-induced mental disorders such as depression, posttraumatic stress disorder (PTSD) and anxiety. Here, we review evidence from both animal models and humans that is increasingly revealing the neurophysiological and neuropsychological mechanisms that underlie stress susceptibility, as well as active mechanisms underlying the resilience phenotype. Ultimately, this growing understanding of the neurobiological mechanisms of resilience should result in the development of novel interventions that specifically target neural circuitry and brain areas that enhance resilience and lead to more effective treatments for stress-induced disorders. Stress resilience can be improved, but the outcomes and effects depend on the type of intervention and the species treated.

## Introduction

**Figure 1:**
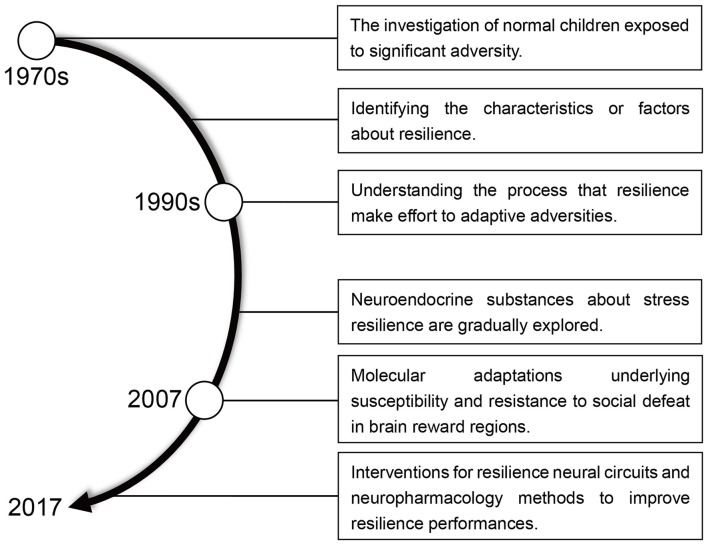
A brief history of resilience research.

Resilience means “the ability to withstand or recover quickly from difficult conditions” (Fletcher and Sarkar, 2013; Robertson et al., 2015). However, in the context of recent biological and psychological research, resilience has gained a more specific meaning. The idea of resilience as resistance to stress (Figure 1) originated in the 1970s when researchers began to study children capable of normal development despite a difficult upbringing (Masten, 2001). By the early 1990s, the emphasis of resilience research has shifted away from identifying protective factors, which involve positive emotions and the competence for self-regulation, to a study of how individuals overcome adversity and an examination of the psychosocial determinants of resilience in trauma-exposed adults (Luthar et al., 2000; Conger and Conger, 2002; Bonanno et al., 2015; Cai et al., 2017). Negative manifestations of resilience manifest as mood disorders, including major depressive disorder (MDD), fear, anxiety, posttraumatic stress disorder (PTSD) and other stress-associated negative emotions (Feder et al., 2009; Friedman, 2014; Alves et al., 2017).

Recent studies employing advanced technologies such as optogenetics have significantly deepened our understanding of the intrinsic biological mechanisms of resilience. This review article will first introduce the psychological and physiological perspectives of resilience, then describe the important neural circuits and neuroendocrine mechanisms involved in resilience, and finally discuss possible ways of improving resilience based on new insights provided by neurobiological studies.

## Fundamental Concepts and Features of Resilience Research

In the last 10 to 15 years, resilience has been examined in a range of contexts in both humans and animals. Animals that show fewer deleterious effects of stress are considered resilient (Steimer and Driscoll, 2005; Krishnan et al., 2007; Feder et al., 2009; Ergang et al., 2015). A number of animal models have been used to improve our understanding of stress resilience or susceptibility (Table 1), for example, chronic social defeat stress (CSDS; Golden et al., 2011), learned helplessness (LH; Berton et al., 2007; Fleshner et al., 2011), exposure to predator odor (Cohen et al., 2012) or chronic mild stress (CMS; Delgado y Palacios et al., 2011). Resilient and susceptible animals can be distinguished by their performance in specific behavioral tasks.

**Table 1:** Research methods or models of stress resilience.

Stress model	Subject	Model overview	References
Chronic social defeat stress (CSDS)	Rodents	Experimental mice are exposed to an aggressive retired breeder CD1 mouse. After interaction the experimental mice are housed in the same cage with a perforated divider separating them from the CD1 mouse. This is a well-established protocol yielding stress-susceptible or resilient cohorts.	Krishnan et al. (2007) and Isingrini et al. (2016)
Chronic restraint stress	Rodents	The rodents are housed in a restrainer with no mobility for a short period daily and then are replaced in their home cage.	Nasca et al. (2015)
Early life stress	Rodents	Pups are separated from their parents during the first postnatal week. Paternal stress and gestational stress operate similarly, but with different separation periods.	Heim and Binder (2012) and Santarelli et al. (2017)
Chronic mild stress (CMS, unpredictable stress)	Rodents	Rodents are exposed to varying physical and psychosocial stresses, for example, shaking, cage tilting.	Suo et al. (2013) and Higuchi et al. (2016)
Learned helplessness (LH)	Rodents	A subset of animals exposed to unavoidable aversive stimuli (e.g., foot shock) develops learned helplessness, i.e., fails to escape when escape available.	Berton et al. (2007) and Brachman et al. (2016)
Use of predator odor	Rodents	Animals are exposed to predator-scent stress.	Cohen et al. (2012)
Acute stress models	Rodents	Breeding of rodent strains with markedly different responses in acute stress environments, such as the tail suspension test. These models are less directly applicable in resilience studies.	El Yacoubi et al. (2003)
Other animal models	Rodents	Comparisons across inbred lines of rats and mice, and selective breeding of rodent lines that display differential stress responses.	Crowley and Lucki (2005) and Overstreet et al. (2005)
Questionnaire investigation	Humans	Investigation of populations to uncover different resilience responses and resilience scale performances.	Hemington et al. (2017)
Psychotherapy or intervention	Humans	Exploration of suitable methods for improving adaptability using resilience interventions.	Langley et al. (2015)
Behavioral experiments and imaging technology	Humans	Evaluation and comparison of brain function while conducting various behavioral tasks relating to stress sensitivity in humans tusing non-invasive imaging technology (such as functional magnetic resonance imaging, fMRI).	Johnson et al. (2014) and Peterson et al. (2014)

Whether resilience should be defined as a trait, process or outcome is frequently debated in human resilience studies. Connor and Davidson (2003) believe that resilience represents personal qualities that enable an individual to thrive in the face of adversity; therefore, in their opinion, resilience is a trait comprising a constellation of characteristics that enable individuals to adapt to the circumstances they encounter (Connor and Davidson, 2003). In contrast, the “process hypothesis” focuses on the interaction between the individual and adverse circumstance and emphasizes that changes over time are dynamic, encompassing positive adaptation within the context of significant adversity (Luthar et al., 2000). Finally, resilience can also be considered an outcome after experienced adversity (Masten, 2001). It is worth noting that all the above concepts of stress resilience have two elements in common, adversity and positive adaptation (Fletcher and Sarkar, 2013), which therefore must both be included in studies of resilience in human and animal models. In fact, most current psychological resilience studies involve four aspects: (a) baseline or pre-adversity; (b) the adversity itself; (c) post-adversity resilient outcomes; and (d) predictors of resilient outcomes; (Bonanno et al., 2015).

More cross-sectional studies that integrate different types of adverse stress are needed to clarify whether different stresses share common influential pathways. This is particularly important given that the term “adversity” covers a wide range of experiences. For example, in humans, adversity can encompass social rejection, failure in examinations, early life stress, depression and other chronic enduring stressful experiences, while in animals it can mean social defeat, forced swimming, foot shock and other types of acutely stressful stimulation (Janakiraman et al., 2016).

Depending on the specific stressful process, resilience might be understood as the ability: (1) to maintain natural functions and elude adversity; and (2) to deal with the stress positively and obtain some benefit from it. Neurobiological studies show that resilience is mediated by both the absence of certain key molecules that occur in susceptible animals and impair their coping ability, and the presence of distinct adaptation mechanisms seen in resilient individuals that promote normal behavior (Krishnan et al., 2007; Friedman et al., 2016). The former and latter are considered to be mechanisms of passive and active resilience, respectively (Russo et al., 2012).

## Representative Animal Models of Resilience

CSDS (Golden et al., 2011) and CMS (Liu et al., 2018) are two of the most widely used resilient animal models and have been widely applied in the study of resilience and depression, although the more aggressive behavior of the outbred CD-1 mouse requires careful monitoring in the CSDS test (Albonetti and Farabollini, 1994). CMS consists of various random negative stressful stimuli, such as foot shock, swimming in cold water, light/dark succession and hunger (Chang and Grace, 2014), and may be more similar to the types of stress experienced by humans. Since female mice exposed to CMS are less stable than males (Franceschelli et al., 2014), gender differences should be taken into account when using this model.

Although such animal models have dramatically improved our understanding of the neural substrates underlying resilience, they have been less useful in defining the complex interactions between environmental stress, protective factors and individual personality. For example, increased self-criticism and decreased self-compassion enhance the risk of depression in humans (Ehret et al., 2015), but these effects are not represented (and arguably could not be represented) in animal models of resilience. On the other hand, techniques used to study regions of the brain involved in the regulation of human resilience, such as functional magnetic resonance imaging (fMRI), positron emission tomography (PET) or deep brain stimulation (DBS), are limited by low spatio-temporal resolution and ethical considerations. Therefore, an appropriate combination of human and animal models is required to enable researchers to gain a precise understanding of resilience.

## Behavioral Characteristics of Resilience

A range of psychosocial factors that contribute to resilience have been identified. The factors include active coping (Snow-Turek et al., 1996; Hanton et al., 2013), optimism (Warner et al., 2012), cognitive reappraisal (Maren, 2008; Farchi and Gidron, 2010; Troy et al., 2010), prosocial behavior (Staub and Vollhardt, 2008), social support and others (Ozbay et al., 2008; Cai et al., 2017). Social support is one of the main protective elements that influence family well-being, parenting quality and child resilience (Armstrong et al., 2005). A 10-year longitudinal study found that social support from partners promoted resilience in response to economic stress (Conger and Conger, 2002). In contrast, poor social support enhanced stress, leading to elevated heart rate (Stansfeld et al., 1997), depression (Oxman and Hull, 2001) and increased susceptibility to PTSD (Johnson et al., 1997).

Although the onset of psychiatric disorders such as PTSD and depression might be prevented by promoting adaptation to stress, the key to resilience and mental well-being lies in emotion regulation processes (Hu et al., 2014). For example, social support and resilience have multiple mediation effects on the regulation of cognitive emotion and acute stress in Chinese male soldiers (Cai et al., 2017). However, there are also studies that claim there are no relationships between resilience and social support, lifestyle factors or work-related factors (Corina and Adriana, 2013; Black et al., 2017), although it is generally acknowledged that resilience buffers against various types of stress. These inconsistent behavioral results might be attributed to the use of different resilience questionnaires, the sample size and type of human subjects, and non-standardization of the test procedure, suggesting that it is critical to identify the physiological substrates underlying the manipulation of resilience. Animal models and emerging technologies, such as optogenetics (Friedman et al., 2014, 2016), electrophysiological recording (Christoffel et al., 2015; Friedman et al., 2016) and animal imaging systems (Delgado y Palacios et al., 2011; Anacker et al., 2016), are generating a great deal of interest in the elucidation of the neural circuits and molecules involved in resilience.

## A Dynamic Framework of Resilience

In early studies, psychological models of resilience were established to describe the construction of active pathways of resilience. Garmezy et al. (1984) emphasized the interaction between adverse stimulation and the consequences of stress, while Rutter (1987) elaborated four pathways to elucidate how individuals process adversity, which involve reduction in risk impact and negative chain reactions, establishment and maintenance of self-esteem and self-efficacy, and the opening up of opportunities. These early theories had a great impact on psychological perspectives of resilience, and subsequently these and other contemporaneous researchers attempted to uncover how resilience interacts with environmental stress and other personal traits to influence the behavior of individuals.

The concept of “biopsychospiritual homeostasis” was introduced by Richardson (2002), whose model proposed that resilience was a dynamic equilibrium state in which physical, psychological and spiritual ingredients, as well as various adversity or protective factors reached a balance. However, Rutter (2012) was forthright in declaring that resilience “should not constitute a theory, nor should it be seen as equivalent to positive psychology or competence.”

Although neurobiological research into resilience has less theoretical underpinning, new discoveries have emerged in recent years. For example, resilient individuals have been shown to have drastically different behavioral performances and neural substrates compared with, more susceptible individuals (Feder et al., 2009), while some recent study shows that a K^+^ channel in ventral tegmental area (VTA) dopamine (DA) neurons differentially mediates neuronal activity in resilient, normal and susceptible mice (Friedman et al., 2016; Han and Nestler, 2017; Barrese et al., 2018).

## Neural Basis of Resilience

Researchers have demonstrated that various brain structures and pathways are involved in resilience (Franklin et al., 2012; Russo et al., 2012), and we review these below.

### Medial Prefrontal Cortex

The medial prefrontal cortex (mPFC) exerts strong negative control over stress pathways, and maladaptive behavior in response to stress involves mPFC dysfunction (Wang et al., 2014). Inhibiting neuronal activity in the mPFC by DBS is effective at alleviating symptoms in depressed humans or rodent depression models (Covington et al., 2010; Warden et al., 2012), while enhanced mPFC excitation results in depression-like behavior (Wang et al., 2014). mPFC lesions augment the hypothalamic-pituitary-adrenal (HPA) axis in response to emotional stress, while, in contrast, intra-mPFC injection of corticosterone attenuates this response (Diorio et al., 1993). Neural activity and levels of immediate early gene expression are lower in the ventral mPFC following stressors such as CSDS, predator stress, or water submersion in susceptible rodents (Covington et al., 2010). Interestingly, depressive patients demonstrate decreased neuronal activity in the postmortem anterior cingulate cortex (ACC), a brain area with functional homology to the mPFC in rodents (Covington et al., 2010). Moreover, hypoactivity is corrected by optogenetic induction of cortical burst firing in animals and is accompanied by the reversal of CSDS-induced social anxiety and anhedonia (Covington et al., 2010; Adamec et al., 2012). In addition, the subgenual cingulate cortex, which is also homologous to the rodent mPFC, is hyperactive in mood disorders (Ressler and Mayberg, 2007; Drevets et al., 2008; Hamani et al., 2011).

The lateral prefrontal cortex often demonstrates hypoactivity in neuroimaging studies of depressed patients (Kinou et al., 2013; Rive et al., 2013). Selective activation of the mPFC–lateral habenula (LHb; Li et al., 2011; Warden et al., 2012) or the mPFC–amygdala pathway (Martinez et al., 2013; Moscarello and LeDoux, 2013) results in depression-like activity. However, stimulation of the mPFC–dorsal raphe nucleus (DRN) pathway promotes resilience (Warden et al., 2012). The precise mechanisms by which the mPFC interacts with its downstream targets and integrates different behavioral responses to stress, and in particular resilience to stress, deserve further investigation.

It should be noted that the results of research into the effect of early life stress on resilience remain inconsistent. Stress has extensively proven to be related to the emergence of diabetes (Marcovecchio and Chiarelli, 2012), child health issues (Charmandari et al., 2012), cardiovascular disease (Kivimäki and Steptoe, 2018) and depression (Pena et al., 2017). Adverse childhood experiences (ACEs), such as psychological or sexual abuse, violence against the mother and household dysfunction, are strongly correlated with a significantly increased risk of physical or psychological disease and unhealthy habits in subsequent life, for example, alcoholism, depression, smoking, severe obesity and illicit drug use (Felitti et al., 1998; Anda et al., 1999; Dube et al., 2001, 2003; Van Niel et al., 2014; Gilbert et al., 2015). Although it is well known that ACEs can result in serious longstanding consequences, mild exposure to stress at an early age, so-called stress inoculation (Meichenbaum and Cameron, 1989), might improve resilience. However, despite investigations into the mechanisms underlying the effects of stress inoculation, which have focused mainly on HPA axis-related variations (reviewed in Ashokan et al., 2016), the potential neural circuits and plasticity have remained elusive. A recent study demonstrated that learned-helplessness mice exposed to inescapable foot shocks for 6 days experienced spatial memory deficits and decreased basolateral amygdala-ventral hippocampus CA1 connection. However, under the same conditions learned-hopefulness mice showed enhanced spatial memory and neural activity (Yang et al., 2016), suggesting the existence of neural plasticity variations associated with a long period of negative stress.

### Hippocampal Pathways

The hippocampus, which is modulated by stress hormones, is one of the main brain areas that exert regulatory control over the HPA axis. Stressors rapidly stimulate the parvocellular neurons of the paraventricular nucleus of the hypothalamus to secrete corticotropin-releasing factor and vasopressin, triggering the release of adrenocorticotropic hormone from the anterior pituitary; in turn the latter promotes the release of glucocorticoid stress hormones from the adrenal cortex into the circulation (Levone et al., 2015). There are both direct and indirect polysynaptic connections between the paraventricular nucleus and the hippocampus, which negatively influence the HPA axis via glucocorticoid-receptor- or mineralocorticoid-receptor-dependent feedback (Franklin et al., 2012; Levone et al., 2015). It has been shown, in both human and rodents, that stimulation of the hippocampus decreases glucocorticoid secretion, while, in contrast, hippocampal lesions increase basal glucocorticoid levels, especially during the stress recovery phase, the phase most reliant on negative feedback (Jankord and Herman, 2008).

The hippocampus is particularly vulnerable to the impact of stress. The glutamate hypothesis, of which impaired hippocampal function is a major component, has been widely accepted in the field of depression. Human studies show that, abnormal glutamatergic synaptic transmission, maladaptive structural and functional changes in hippocampal circuitry, and reduction in hippocampal volume, are associated with stress-induced conditions such as MDD (Franklin et al., 2012). Glutamatergic ventral hippocampus (vHIP) → nucleus accumbens (NAc) projections regulate susceptibility to CSDS. Reduced activity in the vHIP has been observed in mice resilient to CSDS (Bagot et al., 2015). Suppression of vHIP-NAc synaptic transmission by optogenetic induction of long-term depression is pro-resilient, while enhanced activity of this pathway is pro-susceptible. However, optogenetic activation of either mPFC or basolateral amygdala afferents to the NAc is pro-resilient (Bagot et al., 2015), highlighting an important circuit-specific mechanism in depression or stress resilience. Using magnetic resonance imaging, social avoidance in C57BL/6 mice with CSDS correlated positively with volume of the hippocampal CA3, accompanied by synchronized anatomic differences between hippocampus and several other areas, including the VTA, the cingulate cortex and the hypothalamus (Anacker et al., 2016).

Various postsynaptic receptors of the hippocampus, such as G-protein coupled gama-aminobutyric acid B (GABA_B_) receptors, play important roles in stress regulation. Different isoforms of GABA_B_ receptor subunits, such as GABA_B(1a)_ and GABA_B(1b)_, have been shown to differentially regulate stress resilience. Specifically, GABA_B(1a)_ knockout mice are susceptible whereas GABA_B(1b)_-deficient mice are resilient to stress-induced anhedonia and social withdrawal (O’Leary et al., 2014), suggesting that GABA_B_ receptors may be novel therapeutic targets for regulation of stress. Hippocampal serotonin (5-HT) receptors are also important in stress regulation. Thus, knockdown of the hippocampal 5-HT receptor, 5-HT_1A_, significantly decreases the antidepressant-like effect induced by a nicotinic partial agonist, cytisine (Mineur et al., 2015).

### VTA-NAc Pathways

A well-characterized reward circuit in the brain comprises dopaminergic neurons in the VTA that give projections to the NAc. This VTA–NAc circuit is crucial for reward motivation and the consumption of addictive substances (Skibicka et al., 2013; Juarez et al., 2017). However, increasing evidence in humans and animals suggests that VTA-NAc pathways also play an important role in mediating stress susceptibility.

DA neurons in the VTA mediate susceptibility and resilience in CSDS-induced behavioral abnormalities. VTA DA neurons exhibit low frequency tonic firing and high frequency phasic firing *in vivo* (Grace et al., 2003). Induction of phasic, but not tonic, firing by optogenetic stimulation in VTA DA neurons results in a susceptible phenotype in mice undergoing subthreshold CSDS, as indicated by social avoidance and decreased sucrose consumption.

Activation of VTA-NAc, but not VTA-mPFC, pathways leads to stress susceptibility to CSDS, highlighting a circuit-specific mechanism in stress resilience (Razzoli et al., 2011). In support of the above finding, VTA DA neurons of susceptible mice exhibit hyperactivity (Friedman et al., 2014). In contrast, mice resilient to CSDS exhibit stable normal firing of these neurons (Friedman et al., 2014, 2016). mTOR, which has been shown to regulate cell growth, metabolism, proliferation and survival (Elghazi et al., 2017), shows elevated levels in the VTA 3 weeks after termination of CSDS in mice. Levels of phosphorylated AKT, an upstream regulator of mTOR, are also increased (Der-Avakian et al., 2014).

GABAergic medium spiny neurons (MSNs) are the principal neurons in the NAc. Recent studies suggest that impairment of GABAergic neurons in the NAc is linked to MDD. The NAc of stressed mice features a decrease in inhibitory synapses, leading to NAc dysfunction (Zhu et al., 2017). Mice in which the metabotropic glutamate receptor subunit 5 (mGluR5) is deleted display an increase in depression-like behavior compared to controls, while lentiviral transfection of mGluR5 in the NAc of these mutant mice counteracts their depression-like behavior (Shin et al., 2015).

## Towards Improvement of Resilience

Both psychological and behavioral therapy have been used to improve resilience and thus reduce the symptoms of mental disorders and increase mental flexibility (Wolmer et al., 2011; Horn et al., 2016; Creswell, 2017). The drawback of psychological treatments is clear, as behavioral psychotherapy generally takes place over a long period of time, works slowly, and provides little improvement in our understanding of the internal mechanisms involved. Resilience is probably influenced largely by active adaptations, which occur specifically in resilient individuals. Genome-wide screening using the CSDS model has recognized numerous gene expression variations and chromatin alterations in the VTA and NAc that are observed only in resilience (Krishnan et al., 2007; Wilkinson et al., 2009). Thus, it seems possible to trigger natural mechanisms underlying resilience, which differ from the effects of existing antidepressants, in susceptible populations (Russo et al., 2012). We next discuss important research that has changed the understanding of resilience and indicates how new treatments of stress-related disorders might be developed.

### Improving Resilience by Altering Neural Activity

Early studies showed that the degree of VTA DA neuronal activity is a crucial element determining behavioral susceptibility. Thus, *ex vivo* VTA neuronal firing increases in brain tissue of susceptible but not resilient mice (Krishnan et al., 2007; Feder et al., 2009), showing a negative correlation with social avoidance behavior (Cao et al., 2010). Either chronic, but not acute, administration of the antidepressant, fluoxetine, or optogenetic stimulation of VTA DA neurons, completely reverse these deleterious effects in susceptible mice (Cao et al., 2010; Chaudhury et al., 2013). Moreover, the hyperpolarization-activated cation current (I_h_) increases in VTA DA neurons of susceptible mice, while chronic treatment with fluoxetine normalizes increased I_h_ (Cao et al., 2010). Local application or systemic administration of retigabine, a KCNQ-type K^+^ channel opener, normalizes VTA DA neuron hyperactivity and depressive behavior (Friedman et al., 2016), identifying KCNQ as a target for conceptually novel antidepressants or methods of stress regulation. However, an even larger significant increase in I_h_, in parallel with increased K^+^ channel currents, is observed in resilient, compared to susceptible and control mice. Further experimental enhancement of I_h_ or an optogenetic activation of VTA DA neuron activity completely reverses depression-related behavior in susceptible mice (Friedman et al., 2014).

So we might ask, why don’t we observe hyperactivity of VTA DA neurons in resilient mice? One possibility is that the upregulation of I_h_ in VTA DA neurons of resilient mice could drive neuronal firing to extremely high frequencies in parallel with activating a self-tuning K^+^ current mechanism to normalize the excessive firing. The I_h_ potentiation could engender the overactivity that directly causes this K^+^ current compensation (Friedman et al., 2014), a homeostatic plasticity mechanism established in the VTA-NAc, rather than in the VTA-mPFC pathway; these observations might lead to new therapeutic strategies for promoting natural resilience.

Increased activity in the NAc DA1-MSN pathway promotes resilience, while suppression of these MSNs leads to a depression-like phenotype after CSDS. Although bidirectionally modifying the NAc DA2-MSN pathway does not change behavioral outcomes in the CSDS model, repeatedly activating NAc DA2-MSNs evokes social avoidance in resilient mice after subthreshold CSDS (Francis et al., 2015). Therefore, the NAc DA1-MSN pathway may provide novel targets for the treatment of depression or other affective disorders.

Considering the direct anatomical and functional connections between the locus coeruleus (LC) noradrenergic neurons (NEs) and the VTA, the LC might be responsible for buffering the external stressors and stress response of VTA DA neurons (Guiard et al., 2008; Chandler et al., 2013). LC-VTA NE synaptic transmission is both necessary and sufficient for the promotion of resilience in response to social defeat. Furthermore, selective change in NE tone affects VTA DA-NAc projections (Isingrini et al., 2016). Chronic treatment with idazoxan, an α2 NE receptor antagonist, leads to reduction in VTA DA neuron excitability, which counteracts susceptibility (Chaudhury et al., 2013; Isingrini et al., 2016), but whether K^+^ current compensation underlies the decreased neuronal excitability of the VTA DA system requires further investigation.

### Improving Resilience Using Neuropharmacological Approaches

Various neurochemicals have been found to change resilience. The release of NPY, a 36 amino-acid peptide, is thought to help limit the negative consequences of stress and has anxiolytic-like effects (Cohen et al., 2012). Ketamine and a number of other neurochemicals likewise also bring about intense and enduring adaptations to stress (Sachs et al., 2015; Sciolino et al., 2015; Brachman et al., 2016).

#### NPY

Intranasal NPY administration provides neuronal protection when applied immediately prior, or following, exposure to traumatic stress in an animal model. Rats pretreated with intranasal NPY before single prolonged stress (SPS) exposure show less depressive-like and anxiety-like behavior (Serova et al., 2013). Traumatic stress-triggered dysregulation of the HPA axis can be prevented by intranasal NPY, which restores proper negative feedback inhibition within the HPA axis by changing the activity of glucocorticoid receptors (Laukova et al., 2014; Serova et al., 2014). Furthermore, 1 week after SPS exposure, when animals have developed symptoms of PTSD, treatment with intranasal NPY reduces anxiety-like and depressive-like behavior (Serova et al., 2014). These results suggest that NPY holds enormous promise for novel therapeutic approaches to the improvement of resilience, although the mechanism underlying NPY function remains unclear.

#### Ketamine

As an antagonist of the glutamatergic N-methyl-D-aspartate (NMDA) receptor, and an activator of α-amino-3-hydroxy-5-methyl-4-isoxazole propionate (AMPA) receptors, ketamine has rapid and sustained antidepressant effects (Berman et al., 2000; Zarate et al., 2006; Murrough et al., 2013; Price, 2016; McGowan et al., 2017). Ketamine infusion induces rapid reduction in the severity of PTSD and depressive symptoms, thereby improving the overall clinical presentation of PTSD patients (Murrough et al., 2013; Feder et al., 2014). Importantly, ketamine does not give rise, clinically, to significant persistent dissociative symptoms (Feder et al., 2014). A recent study showed that a single dose of ketamine prevents CSDS-induced depressive-like behavior. The effects of ketamine were also confirmed in LH and chronic corticosterone mouse models (Brachman et al., 2016), suggesting that ketamine strengthens resilience and thus might be useful in protecting against stress-induced disorders.

Importantly, ketamine may be clinically most useful if administered in a prophylactic manner, i.e., 1 week before a stressor. In mice undergoing the contextual fear conditioning (CFC) paradigm, administration of prophylactic ketamine for 1 week, but not 1 month or 1 h before CFC, prevents the animal from freezing behavior. However, ketamine treatment following CFC or during extinction does not change subsequent expression of fear (McGowan et al., 2017).

Recently, two Nature articles revealed the mechanism underlying the anti-depression effects of ketamine. Depressive rats were found to have increased bursting activity in the lateral habenula (LHb) due to upregulation of an astroglial potassium channel, Kir4.1 (Cui et al., 2018). Ketamine blocked NMDA-dependent bursting activity in the LHb and reversed depression-like symptoms (Yang et al., 2018), implicating the NMDA receptor and Kir4.1 in the LHb as potential targets for treatment of depression. It should be noted, however, that there is limited clinical use of ketamine due to its psychotogenic side effects and addictive liability. In the CSDS and LH models of depression, it was found that R-ketamine is more potent and shows a longer antidepressant effect than S-ketamine, while S-ketamine, but not R-ketamine, precipitates behavioral abnormalities (Yang et al., 2015). Therefore, unlike S-ketamine, R-ketamine could potentially be used to elicit a sustained and safe antidepressant effect. The antidepressant effect of ketamine requires metabolism of (R,S)-ketamine to (2S,6S; 2R,6R)-hydroxynorketamine (HNK). Moreover, the (2R,6R)-HNK enantiomer shows antidepressant actions in mice that is independent of NMDA receptor inhibition but involves early and persistent activation of AMPA receptors (Zanos et al., 2016). The cortical NMDA receptor complex is heteromultimeric, consisting of two GluN1 and two GluN2 subunits, the latter primarily of the GluN2A and GluN2B isotypes (Monyer et al., 1992). In addition to regulating depression-like behavior, the GluN2B-containing NMDA receptor plays a critical role in mediating the rapid antidepressant effect of ketamine (Miller et al., 2014). More work is required to reveal the details of how the NMDA receptor, the AMPA receptor and ketamine interact, and R-ketamine should be explored further as a potentially more effective and safe antidepressant medicine for improving resilience.

#### 5-HT

5-HT is the neurotransmitter that is most relevant to resilience (Kim et al., 2013). Either 5-HT deficiency in the brain or exposure to psychosocial stress promotes the etiology of depression, anxiety, PTSD and other mood disorders (Sachs et al., 2015). Acute stress is associated with increased 5-HT turnover in the amygdala, NAc and PFC (Feder et al., 2009). Reduced levels of brain 5-HT result in enhanced vulnerability to psychosocial stress and thus reduce the antidepressant effects of fluoxetine following stress exposure in mice (Sachs et al., 2015).

The enteric nervous system, also called the gut brain, is intimately linked with 5-HT and resilience (Foster and McVey Neufeld, 2013). Some metabolites, derived from gut microbes, increase the production of 5-HT in the cells lining the colon (Yano et al., 2015). These cells account for 60% and more than 90% of peripheral 5-HT in mice and humans, respectively (Smith, 2015). In particular, germ-free mice, which lack an intestinal microbiome, have an increased turnover rate of key neurochemicals, including striatal 5-HT, which is associated with anxious behavior, but have significantly decreased levels of 5-HT in blood (Diaz Heijtz et al., 2011; Smith, 2015). Moreover, blood 5-HT levels in these mice can be restored by introducing spore-forming bacteria into the intestine (Diaz Heijtz et al., 2011; Smith, 2015), suggesting that gut microbes may directly or indirectly impact neurotransmitter levels, at least in rodents. However, it remains unclear whether these altered levels of 5-HT in the gut trigger a cascade of molecular events that consequently affect brain activity; the situation in humans requires further investigation.

#### Other Means of Improving Resilience

The neuropeptide galanin and a galanin receptor subtype, GalR1–3, are expressed throughout circuits that mediate stress responses, including the mPFC, DRN, LC, hypothalamus, hippocampus, VTA and amygdala (Hawes and Picciotto, 2004). Exposure to stress decreases time spent in open arm exploration in sedentary rats, but not in those treated chronically with intracerebroventricular galanin or exercised rats which have increased galanin levels in the LC, implicating improved resilience in the latter groups. Increased DA overflow and loss of dendritic spines in the mPFC, observed after stress in sedentary rats, are prevented by both exercise and chronic, intracerebroventricular galanin. Moreover, chronic, but not acute, administration of galanin receptor antagonist M40 blocks the resilience-promoting effects of exercise (Sciolino et al., 2015). These results suggest that increased galanin levels promote resilience at both neural and behavioral levels, and galanin may thus improve stress resilience by regulation of mPFC neural plasticity. Phasic stimulation of VTA DA neurons leads to susceptibility and reverses resilience rapidly (Chaudhury et al., 2013), while midbrain DA activity and DA release can be inhibited by galanin (Sciolino et al., 2015; Weinshenker and Holmes, 2016).

#### Improving Resilience in Humans

Improvements in resilience in humans have been reported as a result of psychological and cognitive therapies, such as child caregiver advocacy resilience (Li et al., 2017), a life skills education-based program (Sarkar et al., 2017), the iNEAR programme (Tunariu et al., 2017), intensive mindfulness meditation training (Hwang et al., 2018) and stress inoculation training (Horn et al., 2016). Although all the above achieved good outcomes, the same method may have different therapeutic effects in different individuals. Therefore, the development of more general, stable, and faster effective interventions is likely to be a trend in the future.

DBS is now a well-established surgical option. More and more studies indicate that DBS has beneficial effects in many psychiatric disorders, such as PTSD (Koek et al., 2014), depression (Schlaepfer et al., 2008) and Parkinson’s disease (Pellaprat et al., 2014). Early research identified another clinical tool, repetitive transcranial magnetic stimulation (rTMS), a non-invasive technique that normalizes activity of the HPA system and has an antidepressant effect (Czéh et al., 2002). It seems that rTMS induces alterations in neural networks and has an effect in some psychiatric disorders (Aleman, 2013). Since resilience is closely related to these diseases, it is possible that these technologies can be used to improve resilience, but this remains to be studied.

## Concluding Remarks

The enormous impact of stress, trauma or other forms of adversity on humanity, together with limitations in available treatments, make it necessary to explore resilience mechanisms that might protect against PTSD, depression and other mental disorders. Most work in this discipline over the past decades has focused on the biological differences between resilience and susceptibility, and has explored, in animal models, means to reverse the deleterious effects of chronic stress. However, reversing these deleterious effects does not necessarily mean that resilience is enhanced and that the affected individuals have a better life. A crucial novel perspective, which has emerged in recent years, is that resilient animals have active adaptive mechanisms that are distinct from actions that reverse deleterious effects in susceptible animals. Therefore, current research aimed at improving stress resilience focuses on the relationship and essential distinction between reversing deleterious effects and cultivating active adaptive mechanisms. Finally, the development of “precision medicine” for improving stress resilience will require a clearer picture to emerge out of the messy realm of current resilience research. A dynamic and integrated combination of psychological and neurobiological studies will be essential for generating this clearer picture of resilience. Further studies should focus not only on the resilience of individuals or small human/animal populations, but also the wider human community, much of which is under pressure due to, for example, the global economic crisis. Given that social support, economic pressure and prosocial behaviors have a significant influence on an individual’s response to stress, it will be necessary to uncover neuronal and psychological mechanisms of resilience at the level of different human communities.

Although mild exposure to stress at an early age (stress inoculation; Meichenbaum and Cameron, 1989), might improve resilience, it is worth noting that ACEs, at high score, have been proven to affect brain development, resulting in enormous, awful and long-term sequelae in adulthood (Felitti et al., 1998; Anda et al., 1999; Van Niel et al., 2014; Gilbert et al., 2015). Importantly, the consequence of ACEs is longstanding due, at least in part, to DNA methylation changes of BDNF gene (Kundakovic et al., 2015). Therefore, efforts toward reducing the childhood trauma that may require a public health campaign would have the greatest impact for the prevention of ACEs.

## Author Contributions

HL and LY designed the theme of the manuscript and wrote most sections. CZ and YJ together wrote one section. All authors approved the manuscript for submission and publication.

## Conflict of Interest Statement

The authors declare that the research was conducted in the absence of any commercial or financial relationships that could be construed as a potential conflict of interest.
